# Risk factors for asthma exacerbation during pregnancy: protocol for a systematic review and meta-analysis

**DOI:** 10.1186/s13643-022-01975-8

**Published:** 2022-06-07

**Authors:** Marleen P. Bokern, Annelies L. Robijn, Megan E. Jensen, Daniel Barker, Katherine J. Baines, Vanessa E. Murphy

**Affiliations:** 1 Department of Pharmaco-Therapy, Epidemiology and Economics, University of Groningen, Groningen, The Netherlands; 2 Priority Research Centre for Healthy Lungs, University of Newcastle, New Lambton Heights, NSW Australia; 3 School of Medicine and Public Health, University of Newcastle, Newcastle, NSW Australia; 4 Hunter Medical Research Institute, University of Newcastle, Level 2 West Wing, University Drive, Callaghan, NSW 2308 Australia

**Keywords:** Asthma, Pregnancy, Risk factors

## Abstract

**Background:**

Asthma is the most common medical condition to affect pregnancy. Asthma exacerbations occur in up to 45% of pregnant women and have been associated with adverse perinatal and infant outcomes. Conflicting literature exists regarding the risk factors for exacerbations, and no synthesis of the literature currently exists. Therefore, this systematic review and meta-analysis aims to determine risk factors for asthma exacerbations during pregnancy among pregnant women with asthma.

**Methods:**

This protocol has been reported according to the Preferred Reporting Items for Systematic Review and Meta-Analysis protocols checklist. A systematic search will be conducted in the electronic MEDLINE, Embase, CINAHL and Cochrane Clinical Trials Register databases (from January 2000 onwards). Eligibility of each publication will be determined based on predefined selection criteria. Prospective cohort studies, retrospective cohort studies, case-control studies and randomised controlled trials (RCTs) will be included. Quality of included studies will be determined using the Newcastle Ottawa Scale and the Cochrane Risk of Bias tool. Pooled relative risk will be computed using random-effects meta-analyses. Heterogeneity will be assessed using the chi-squared test and the *I*^2^ parameter. Publication bias will be assessed by inspecting a funnel plot for asymmetry and with the Egger’s test of analyses including ten studies or more.

**Discussion:**

The results of this systematic review and meta-analysis will discuss the potential risk factors for asthma exacerbations during pregnancy. This may aid healthcare professionals in early identification of pregnant women with asthma at risk of poor outcomes, providing the opportunity to implement early interventions in order to avoid deterioration of asthma symptoms during pregnancy.

**Systematic review registration:**

PROSPERO CRD42020196190

**Supplementary Information:**

The online version contains supplementary material available at 10.1186/s13643-022-01975-8.

## Background

Asthma is the most common medical condition in pregnancy, affecting about 12% of pregnant women in Australia. Pregnant women with asthma may experience worsening or improving asthma or may have no changes to their condition. Up to 45% of pregnant women with asthma have an asthma exacerbation requiring medical intervention during pregnancy [1]. Uncontrolled asthma and asthma exacerbations have been associated with increased risk of several adverse perinatal outcomes, such as spontaneous abortion [2], preeclampsia [3], and low birth weight [4]. Furthermore, uncontrolled asthma and exacerbations have been associated with poor infant respiratory health, including the development of asthma [3, 5, 6].

Several studies have identified patient-related risk factors for asthma exacerbations during pregnancy, such as maternal age [3, 7], smoking [2, 3, 8, 9], obesity [9–11] and anxiety and/or depression [9, 12]. Other studies have identified disease-related factors such as increasing asthma severity [1, 9, 13–15], lung function [14] and respiratory viral infections [1]. The risks of these factors vary between studies and other studies report no associations.

A Danish study reported that clinically stable asthma, no exacerbation history and no prescription of ICS were associated with a decreased risk of exacerbations during pregnancy [16]. Another Danish study utilising the same study population reported excessive gestational weight gain in the first trimester as an important risk factor for asthma exacerbations [17]. The data on the impact of foetal sex on asthma exacerbations in pregnancy is conflicting with some studies indicating an increased risk when carrying a female foetus [18–20], whereas other studies report no association [3, 21, 22]. The majority of studies reporting risk factors of asthma exacerbations include relatively small study populations; therefore, it is important to combine all available studies in meta-analyses to determine pooled associations between the risk factors and asthma exacerbations.

Given the association between asthma exacerbations in pregnancy and adverse perinatal and infant health outcomes, it is important to synthesise recent literature to identify risk factors which may be modifiable or allow early identification and additional monitoring of women at risk. Therefore, the aim of this systematic review and meta-analysis is to determine risk factors for asthma exacerbations during pregnancy among pregnant women with asthma.

## Methods

This protocol has been registered with PROSPERO international prospective register of systematic reviews (registration number CRD42020196190) and has been reported following the Preferred Reporting Items for Systematic Reviews and Meta-Analysis Protocol (PRISMA-P) [23]. The final review will be reported following the PRISMA statement [24] and the Meta-Analysis of Observational Studies in Epidemiology (MOOSE) guidelines [25].

### Information sources and search strategy

We developed our search strategy with the support of a research and scholarly communication advisor. A search will be conducted of the electronic MEDLINE, Embase, CINAHL and Cochrane Clinical Trials Register databases to identify articles for possible inclusion. The search terms with multipurpose indicators (asthma or wheeze) and (pregnan* or perinat* or obstet*) and (exacerb* or flare up or morbidit* or attack*) will be used to identify all potential studies for inclusion. The search will be limited to include studies conducted from 2000 to the present time to keep the review relevant to contemporary research. Only studies available as an English language publication will be considered for inclusion, due to limited resources for language translation. The reference lists of included studies will be searched by hand to identify cited articles not identified by the electronic search (Additional file 1).

### Study selection

Two independent reviewers will screen the abstracts of all identified studies. Studies that are potential articles for inclusion in the review will be obtained in full-text for assessment by the same reviewers. Any disagreements at either stage of study selection will be resolved by consensus or referral to a third reviewer. Studies rejected at each stage of the review will be recorded, along with the reasons for their rejection at the full-text screening stage. The selection of studies will be performed using Covidence [26], a software designed for conducting systematic reviews.

### Eligibility

Studies will be selected based on the following criteria of study population, exposure and outcome of interest. Studies published in English from the year 2000 will be included, to keep the review relevant to contemporary research (20 years look back period from protocol development time). Prospective cohort studies, retrospective cohort studies, case-control studies and randomised controlled trials (RCTs) will be included.

We will include studies that contain data from pregnant women with a diagnosis of asthma. Maternal asthma could be defined as physician-diagnosed (whether confirmed or subject self-report) or database-coded asthma diagnosis.

The main exposure to review will be patient characteristics that may act as risk factors/predictors for exacerbation. These could include, but are not limited to, demographic characteristics (e.g. age, race/ethnicity), characteristics relating to the patients’ asthma (e.g. severity, history, medications), other health-related characteristics (e.g. smoking, body mass index (BMI), mental health, co-morbidities, concomitant medication use, genetics) or characteristics of the pregnancy (e.g. foetal sex, antenatal care type, parity, gestational weight gain).

The outcome will be asthma exacerbations, defined as exacerbations requiring medical intervention such as hospitalisations, emergency department (ED) visits, unscheduled physician visits or oral corticosteroid (OCS) courses for asthma during pregnancy.

### Data extraction

All studies that meet the inclusion criteria at the full-text stage will have the following data extracted (where available) and recorded in a standardised Microsoft Excel form (Microsoft Corporation 2018):

- Study characteristics:
  - ◦ Authors, year of publication, journal, country and study design
  - ◦ Inclusion and exclusion criteria
- Data source and time period of data collection
- Exacerbation definition
- Population characteristics by outcome status
  - ◦ Maternal age
  - ◦ BMI
  - ◦ Smoking status
  - ◦ Ethnicity
  - ◦ Socio-economic status
  - ◦ Comorbidities/co-medication (not including asthma medication)
  - ◦ Antenatal care
  - ◦ Gestational age at recruitment
  - ◦ Gestational weight gain
  - ◦ Parity
  - ◦ Singleton/multiple pregnancy
  - ◦ Foetal sex
- Asthma characteristics by outcome status
  - ◦ Pre-pregnancy asthma history (prior exacerbations)
  - ◦ Asthma severity
  - ◦ Asthma symptoms or control
  - ◦ Asthma management skills
  - ◦ Asthma medication

Data extraction will be performed by one reviewer and checked by a second reviewer, a very thorough process where every detail, including missed details, is scrutinised, consistent with previously published systematic reviews [27–30]. Discrepancies will be noted and discussed by the reviewers until consensus is reached or after discussion with a third reviewer.

### Risk of bias assessment

Included studies will be assessed using the Newcastle Ottawa Scale (NOS) [31] independently by two reviewers for observational studies and the Cochrane Risk of Bias (RoB) tool [32] will be used for RCTs. Quality scores and RoB judgement will be recorded along with the outcomes from data extraction. GRADE (Grading of Recommendations, Assessment, Development and Evaluations) will be used to determine certainty of the meta-analysis results [33, 34].

### Data synthesis and analysis

We will provide a narrative synthesis of the findings from the included studies, structured around subject characteristics and distribution of potential predictors and outcomes. Meta-analyses will be conducted for risk factors reported by two or more studies with comparable exacerbation definitions. The meta-analyses will follow standard methodological guidelines to minimise inherent difficulties caused by the diversity of individual studies based on population characteristics, study designs and measured outcomes. STATA 16.1 (StataCorp College Station, TX, USA) will be used to calculate the relative risk of exacerbations for women with and without potential risk factors. A random effects model will be used for dichotomous outcomes to calculate relative risk with a 95% confidence interval when greater than three studies are combined; alternatively, a fixed effect model will be used. For continuous outcomes, mean difference will be calculated. For studies reporting adjusted estimates, we will extract these estimates along with the adjusting factors and, where possible, combine the adjusted estimates using the generic inverse variance method.

Heterogeneity between studies will be assessed using the chi-squared test (with *P*>0.1 indicating significant heterogeneity), *I*^2^ parameter (where I^2^ > 60% indicates heterogeneity). Sub-analysis by active asthma management will be conducted for studies with significant heterogeneity.

Publication bias will be assessed by inspecting a funnel plot for asymmetry and with the Egger’s test for analyses including ten studies or more. Potential publication bias will be considered present if *p*<0.1.

## Discussion

To our knowledge, this systematic review and meta-analysis will be the first to synthesise the literature on risk factors for asthma exacerbations during pregnancy. The results of this review have the potential to improve antenatal asthma care for women with asthma who are at risk of having an exacerbation during pregnancy, by allowing early identification and increased monitoring or management of asthma. Reducing the risk of these exacerbations may improve perinatal outcomes and infant health.

This proposed review has a number of strengths. Firstly, we will be using established guidelines and bias assessment tools (PRISMA, MOOSE, NOS, the Cochrane Risk of Bias tool and GRADE) to report our findings and assess the quality of included studies. Secondly, we will primarily focus on clinically relevant risk factors of asthma exacerbations such as obesity, smoking status, mental health and asthma severity. Information on these factors is routinely collected at antenatal visits; therefore, the results of this review may assist healthcare providers in identifying pregnant women with asthma who are at risk of having an exacerbation without having to collect additional information or perform any additional tests. The results of this systematic review and meta-analysis may inform future research into reducing the risk of asthma exacerbations during pregnancy. This review may form the basis of a clinical trial addressing a treatable traits approach in order to reduce asthma exacerbations during pregnancy.

There is potential for high heterogeneity as a result of including all pregnant women with asthma regardless of doctor diagnosis or severity from any country. Limiting our search strategy to English language articles is a potential limitation. However, a previous systematic review [27] indicates that the amount of information missed due to language is likely to be very low.

Results of this systematic review and meta-analysis will be presented at conferences and published in a relevant, peer-reviewed journal.

## Supplementary Information

**Additional file 1.** Search strategy example.

## Data Availability

Not applicable.

## Abbreviations

BMI: Body mass index
ED: Emergency department
MOOSE: Meta-Analysis of Observational Studies in Epidemiology
NOS: Newcastle Ottawa Scale
OCS: Oral corticosteroids
PRISMA: Preferred Reporting Items for Systematic Reviews and Meta-Analysis
RCT: Randomised controlled trials

## References

[1] Murphy VE, Gibson P, Talbot PI, Clifton V (2005). Severe asthma exacerbations during pregnancy. Obstet Gynecol.

[2] Yland JJ, Bateman BT, Huybrechts KF, Brill G, Schatz MX, Wurst KE (2020). Perinatal outcomes associated with maternal asthma and its severity and control during pregnancy. J Allergy Clin Immunol Pract.

[3] Abdullah K, Zhu J, Gershon A, Dell S, To T. Effect of asthma exacerbation during pregnancy in women with asthma: a population-based cohort study. Eur Respir J. 2019;55. 10.1183/13993003.01335-2019. 10.1183/13993003.01335-2019 31772000

[4] Namazy JA, Murphy VE, Powell H, Gibson PG, Chambers C, Schatz M (2013). Effects of asthma severity, exacerbations and oral corticosteroids on perinatal outcomes. Eur Respir J.

[5] Martel MJ, Rey É, Beauchesne MF, Malo JL, Perreault S, Forget A (2009). Control and severity of asthma during pregnancy are associated with asthma incidence in offspring: two-stage case-control study. Eur Respir J.

[6] Liu X, Agerbo E, Schlünssen V, Wright RJ, Li J, Munk-Olsen T (2018). Maternal asthma severity and control during pregnancy and risk of offspring asthma. J Allergy Clin Immunol.

[7] Grzeskowiak LE, Smith B, Roy A, Dekker GA, Clifton VL (2016). Patterns, predictors and outcomes of asthma control and exacerbations during pregnancy: a prospective cohort study. ERJ Open Res.

[8] Murphy VE, Clifton VL, Gibson PG (2010). The effect of cigarette smoking on asthma control during exacerbations in pregnant women. Thorax.

[9] Robijn AL, Brew BK, Jensen ME, Rejnö G, Lundholm C, Murphy VE (2020). Effect of maternal asthma exacerbations on perinatal outcomes: a population-based study. ERJ Open Res.

[10] Murphy VE, Jensen ME, Powell H, Gibson PG (2017). Influence of maternal body mass index and macrophage activation on asthma exacerbations in pregnancy. J Allergy Clin Immunol Pract.

[11] Hendler I, Schatz M, Momirova V, Wise R, Landon M, Mabie W (2006). Association of obesity with pulmonary and nonpulmonary complications of pregnancy in asthmatic women. Obstet Gynecol.

[12] Powell H, McCaffery K, Murphy VE, Hensley MJ, Clifton VL, Giles W (2013). Psychosocial variables are related to future exacerbation risk and perinatal outcomes in pregnant women with asthma. J Asthma.

[13] Williams DA (1967). Asthma and pregnancy. Acta Allergol.

[14] Schatz M, Dombrowski MP, Wise R, Thom EA, Landon M, Mabie W (2003). Asthma morbidity during pregnancy can be predicted by severity classification. J Allergy Clin Immunol.

[15] Belanger K, Hellenbrand M, Holford T, Bracken M (2010). Effect of pregnancy on maternal asthma symptoms and medication use. Obstet Gynecol.

[16] Ali Z, Nilas L, Ulrik CS (2018). Determinants of low risk of asthma exacerbation during pregnancy. Clin Exp Allergy.

[17] Ali Z, Nilas L, Ulrik CS (2018). Excessive gestational weight gain in first trimester is a risk factor for exacerbation of asthma during pregnancy: a prospective study of 1283 pregnancies. J Allergy Clin Immunol.

[18] Bakhireva LN, Schatz M, Jones KL, Tucker CM, Slymen DJ, Klonoff-Cohen HS (2008). Fetal sex and maternal asthma control in pregnancy. J Asthma.

[19] Kwon HL, Belanger K, Holford TR, Bracken MB (2006). Effect of fetal sex on airway lability in pregnant women with asthma. Am J Epidemiol.

[20] Beecroft N, Cochrane GM, Milburn HJ (1998). Effect of sex of fetus on asthma during pregnancy: Blind prospective stud. BMJ.

[21] Firoozi F, Ducharme FM, Lemière C, Beauchesne MF, Perreault S, Forget A (2009). Effect of fetal gender on maternal asthma exacerbations in pregnant asthmatic women. Respir Med.

[22] Baibergenova A, Thabane L, Akhtar-Danesh N, Levine M, Gafni A (2006). Is fetal gender associated with emergency department visits for asthma during pregnancy?. J Asthma.

[23] Moher D, Shamseer L, Clarke M, Ghersi D, Liberati A, Petticrew M (2015). Preferred reporting items for systematic review and meta-analysis protocols (PRISMA-P) 2015 statement. Syst Rev.

[24] Moher D, Liberati A, Tetzlaff J, Altman DG. Preferred reporting items for systematic reviews and meta-analyses: the PRISMA statement. BMJ 2009;339:b2535–b2535. doi:10.1136/bmj.b2535. 10.1136/bmj.b2535 PMC2714657 19622551

[25] Stroup DF (2000). Meta-analysis of observational studies in epidemiology: a proposal for reporting. JAMA.

[26] Veritas Health Innovation. Covidence systematic review software. www.covidence.org.

[27] Robijn AL, Jensen ME, McLaughlin K, Gibson PG, Murphy VE (2019). Inhaled corticosteroid use during pregnancy among women with asthma: a systematic review and meta-analysis. Clin Exp Allergy.

[28] Murphy V, Namazy J, Powell H, Schatz M, Chambers C, Attia J (2011). A meta-analysis of adverse perinatal outcomes in women with asthma. BJOG.

[29] Zairina E, Stewart K, Abramson MJ, George J (2014). The effectiveness of non-pharmacological healthcare interventions for asthma management during pregnancy: a systematic review. BMC Pulm Med.

[30] Essat M, Harnan S, Gomersall T, Tappenden P, Wong R, Pavord I (2016). Fractional exhaled nitric oxide for the management of asthma in adults: a systematic review. Eur Respir J.

[31] Wells GA, Shea B, O’Connel D (2009). The Newcastle-Ottawa scale (NOS) for assessing the quailty of nonrandomised studies in meta-analyses.

[32] Higgins JPT, Altman DG, Gotzsche PC, Juni P, Moher D, Oxman AD (2011). The Cochrane Collaboration’s tool for assessing risk of bias in randomised trials. BMJ.

[33] Guyatt GH, Oxman AD, Vist GE, Kunz R, Falck-Ytter Y, Alonso-Coello P (2008). GRADE: an emerging consensus on rating quality of evidence and strength of recommendations. BMJ.

[34] Foroutan F, Guyatt G, Zuk V, Vandvik PO, Alba AC, Mustafa R (2020). GRADE Guidelines 28: Use of GRADE for the assessment of evidence about prognostic factors: rating certainty in identification of groups of patients with different absolute risks. J Clin Epidemiol.

