# Supplementary material for: Risk factors for asthma exacerbation during pregnancy: protocol for a systematic review and meta-analysis
Source: Syst Rev. 2022 Jun 7;11:115. doi: 10.1186/s13643-022-01975-8 (PMC9172055; doi:10.1186/s13643-022-01975-8)
Supplement: Supplementary file 1 — Additional file 1. Search strategy example. [file 13643_2022_1975_MOESM1_ESM.docx]

Risk factors for asthma exacerbation during pregnancy: systematic review and meta-analysis protocol

Marleen P. Bokern, Annelies L. Robijn, Megan E. Jensen, Daniel Barker, Katherine J. Baines, Vanessa E. Murphy

| EMBASE and MEDLINE | |
| --- | --- |
| 1. | asthma.mp. [mp=ti, ab, hw, tn, ot, dm, mf, dv, kw, fx, dq, nm, kf, ox, px, rx, ui, sy] |
| 2. | wheeze.mp. [mp=ti, ab, hw, tn, ot, dm, mf, dv, kw, fx, dq, nm, kf, ox, px, rx, ui, sy] |
| 3. | 1 or 2 |
| 4. | pregnan*.mp. [mp=ti, ab, hw, tn, ot, dm, mf, dv, kw, fx, dq, nm, kf, ox, px, rx, ui, sy] |
| 5. | perinat*.mp. [mp=ti, ab, hw, tn, ot, dm, mf, dv, kw, fx, dq, nm, kf, ox, px, rx, ui, sy] |
| 6. | obstet*.mp. [mp=ti, ab, hw, tn, ot, dm, mf, dv, kw, fx, dq, nm, kf, ox, px, rx, ui, sy] |
| 7. | 4 or 5 or 6 |
| 8. | exacerb*.mp. [mp=ti, ab, hw, tn, ot, dm, mf, dv, kw, fx, dq, nm, kf, ox, px, rx, ui, sy] |
| 9. | flare up.mp. [mp=ti, ab, hw, tn, ot, dm, mf, dv, kw, fx, dq, nm, kf, ox, px, rx, ui, sy] |
| 10. | morbidit*.mp. [mp=ti, ab, hw, tn, ot, dm, mf, dv, kw, fx, dq, nm, kf, ox, px, rx, ui, sy] |
| 11. | attack*.mp. [mp=ti, ab, hw, tn, ot, dm, mf, dv, kw, fx, dq, nm, kf, ox, px, rx, ui, sy] |
| 12. | 8 or 9 or 10 or 11 |
| 13. | 3 and 7 and 12 |
| 14. | limit 13 to english language |
| 15. | limit 14 to yr="2000 -Current" |

Additional file 1. Search strategy example
